# A feasibility study of applying two-dimensional photogrammetry for screening and monitoring of patients with adolescent idiopathic scoliosis in clinical practice

**DOI:** 10.1038/s41598-023-41267-2

**Published:** 2023-08-31

**Authors:** Qian Zheng, Lingfeng Xie, Jiang Xu, Nan Xia, Christina Zong-Hao Ma

**Affiliations:** 1 Department of Rehabilitation Medicine, Tongji Hospital, Tongji Medical College, Huazhong University of Science and Technology, Jiefang Avenue, Wuhan, 430030 China; 2 Department of Biomedical Engineering, The Hong Kong Polytechnic University, Hung Hom, 999077 Hong Kong SAR China; 3 Research Institute for Smart Aging, The Hong Kong Polytechnic University, Hung Hom, 999077 Hong Kong SAR China

**Keywords:** Health care, Medical research

## Abstract

Standing posteroanterior radiographs have been the golden standard to quantify the severity of scoliosis deformity. However, it exposes ionizing radiation to scoliosis patients, and cannot be used for routine screening and monitoring. This study aimed to develop a protocol of measuring postural indexes by using the noninvasive and radiation-free two-dimensional (2D) photogrammetry method and identify its clinical value in scoliosis screening and monitoring. The five postural indexes were measured from the posterior view of 110 participants. One-way ANOVA with post hoc Tukey HSD/Games–Howell analysis was used to compare the differences between the participants in the scoliosis group and the non-scoliosis group. Pearson coefficients of correlation were analyzed to identify the relationships between Cobb angles and each of the five quantitative postural indexes. Based on 2D photogrammetry, the postural indexes of C7 deviation (*p* = 0.02), shoulder alignment (*p* < 0.001), scapula alignment (*p* < 0.001), waist angle discrepancy (*p* < 0.001), and PSIS alignment (*p* < 0.001) could significantly differentiate scoliosis and non-scoliosis patients during screening. The waist angle discrepancy (r = 0.4, *p* = 0.01; r = 0.8, *p* = 0.03; r = 0.7, *p* = 0.01) and shoulder alignment (r = 0.6, *p* = 0.03) had moderate to strong positive correlations with the Cobb angles, which supported their clinical values in monitoring scoliotic curvature changes of adolescent idiopathic scoliosis (AIS) patients.

## Introduction

Adolescent idiopathic scoliosis (AIS) is a complex three-dimensional (3D) structural spinal deformity at the frontal, sagittal, and horizontal planes that occurs in patients in or around the pubertal growth spurt period^1^. The AIS affects approximately 2.4–5.1% of children, and female patients are more prone to develop progressive curves than males^2^.

It is important to provide early detection and regular scoliosis screening, to facilitate the provision of timely treatment at an early treatable stage in AIS patients^3^. Among the various equipment and technologies to achieve this purpose, the measurement of the Cobb angle (i.e. the angle of the spinal curvature between the two most tilted end vertebrae of a scoliotic curve) on standing posteroanterior radiographs has been the golden standard method to quantify the severity of the spinal deformity^4^. However, such radiograph exposes ionizing radiation and hazards to AIS patients^3^, who need to undertake regular monitoring assessments every 4 to 6 months and 6 to 12 months during and beyond the rapid growth phases, respectively^5^.

To reduce radiographic exposure in AIS patients, a growing number of radiation-free assessment methods/technologies; including the scoliometer^6^, the trunk aesthetic clinical evaluation tool (TRACE)^7^, the ultrasound assessment^8, 9^, the surface topography (ST) analysis^10^, the 3D surface topography^11^ and the two-dimensional (2D) photogrammetry method^12–14^, etc.; have been developed and applied in clinical assessment, to supplement the traditional radiographic assessment of AIS patients more recently. Among them, the 2D photogrammetry method can generalize several parameters related to the AIS patients’ postural characteristics and appearance. It is performed by calculating the angles and distances among various anatomical landmarks on the captured photographs^15^. As a quick, easy, inexpensive, and accessible tool, it has received increasing attention in recent years^16^. Previous studies have supported the good reproducibility and reliability level of the 2D photogrammetry method^17^, and the moderate to high correlation between it and the 3D surface topography method in assessing shoulder elevation, pelvis tilt, the deviation of the trunk, and thoracic scoliosis angle^16^.

The 2D photogrammetry method for scoliosis screening and monitoring had also been studied in recent years^13, 14^. Penha et al.^14^ found that the shoulder obliquity was the postural variable associated with adolescent idiopathic scoliosis and should be considered for inclusion in the routines used to assess scoliosis in adolescents in clinical and scholastic environments. However, the insufficient control of the AIS participants’ scoliotic curvature types has been existing in this study^14^, resulting in a lack of the representativeness of the shoulder obliquity index measured by the 2D photogrammetry method for scoliosis screening. Since scoliosis is associated with 3D morphologic modifications of the trunk^11^, the trunk posture indexes of different scoliotic curvature types vary among patients with scoliosis, e.g., the position of the first thoracic vertebra according to the central sacral line among the three curves, four curves and non three-non four curves in the Rigo classification are different^18^.

Bago et al.^13^ found that clinical photography is a valid method for assessing trunk asymmetry in severe idiopathic scoliosis (i.e., Cobb degrees range 40°–101°), and specifically for waist area measurements, the robust cutoff values can be determined to discriminate among different curve patterns according to Lenke classification. However, the insufficient control of the confounding factor (i.e., the leg length discrepancies) and the limitation of the included severe idiopathic scoliosis candidates (i.e., Cobb degrees range 40°–101°) have existed in this study^13^, influencing the accuracy and the application range of the waist area measurements and robust cutoff values postural indexes measured by the 2D photogrammetry method for scoliosis monitoring. For example, a severe leg-length discrepancy (i.e., the difference in leg lengths is > 20 mm) can result in lumbar scoliosis and other postural defects^19, 20^.

Thus, more in-depth study with improved control of the AIS participants’ homogeneity and the confounding factors are needed to address the clinical need. Meanwhile, the potential application of this technology in AIS patients with mild to moderate scoliotic curvature (i.e., Cobb degrees between 10° and 45°)^1^ and receiving the conservative treatment has also remained unclear. To address the above-mentioned issues, the application of 2D photogrammetry method in assessing the postural indexes for screening and monitoring of AIS patients with mild to moderate scoliotic curvature and receiving the conservative treatment, with improved control of patients’ homogeneity and confounding factors, in the clinical practice will be investigated in this study.

In summary, this study aimed to: (1) identify several postural indexes that could be used to screen and identify whether a AIS patient’s Cobb angle is larger than 10° or not; and (2) investigate the relationship between the postural indexes and Cobb angles; in AIS patients with mild to moderate curvatures (i.e., Cobb degrees between 10° and 45°) from the single-curve/C-shape group and the double-curve/S-shape group, by improving the control of the confounding factor (i.e., leg length discrepancies) of measuring postural indexes. The findings of this study can build on the evidence regarding the application of the 2D photogrammetry in screening and monitoring of scoliotic curvature in the AIS patients with mild to moderate curvatures (i.e., Cobb degrees between 10° and 45°) and receiving conservative treatments.

## Materials and methods

### Participants

The participants were retrospectively recruited from the outpatient clinic (specializing in the treatment of scoliosis) from July 2016 to June 2023. The inclusion criteria of participants were: (1) scoliosis patients diagnosed as adolescent idiopathic scoliosis (Cobb angle between 10° and 45°) with the assessment results of the standing posteroanterior full-spine radiographs, and non-scoliosis participants (angle of trunk rotation (ATR) appearing in the Adam’s test measured by the Scoliometer < 4°^21^); (2) 10–16 years old; (3) no prior treatment; and (4) with the assessments results of the 2D photogrammetry of the posterior view of the whole body in standing position^14^. The participants with additional diseases that may cause abnormalities in the musculoskeletal system, or with leg length discrepancies were excluded from this study^22^.

According to the definition of Scoliosis Research Society, the AIS patients shall be patients aged between 10 and 17 years old with a Cobb angle above 10°^23^. Based on this definition, the AIS participants were further divided into three groups: (1) single-curve scoliosis group, (2) double-curve scoliosis group, and (3) Non-scoliosis group.

This study was conducted in accordance with the Declaration of Helsinki, and was approved by the Medical Ethics Committee of Tongji Medical College, Huazhong University of Science and Technology (protocol code: [2020] (S173); date of approval: 8th Jul 2020). The informed consents were obtained from the AIS participant, whose photos were used in this paper, and his parents by telephone consultation.

### Equipment

**Figure 1:**
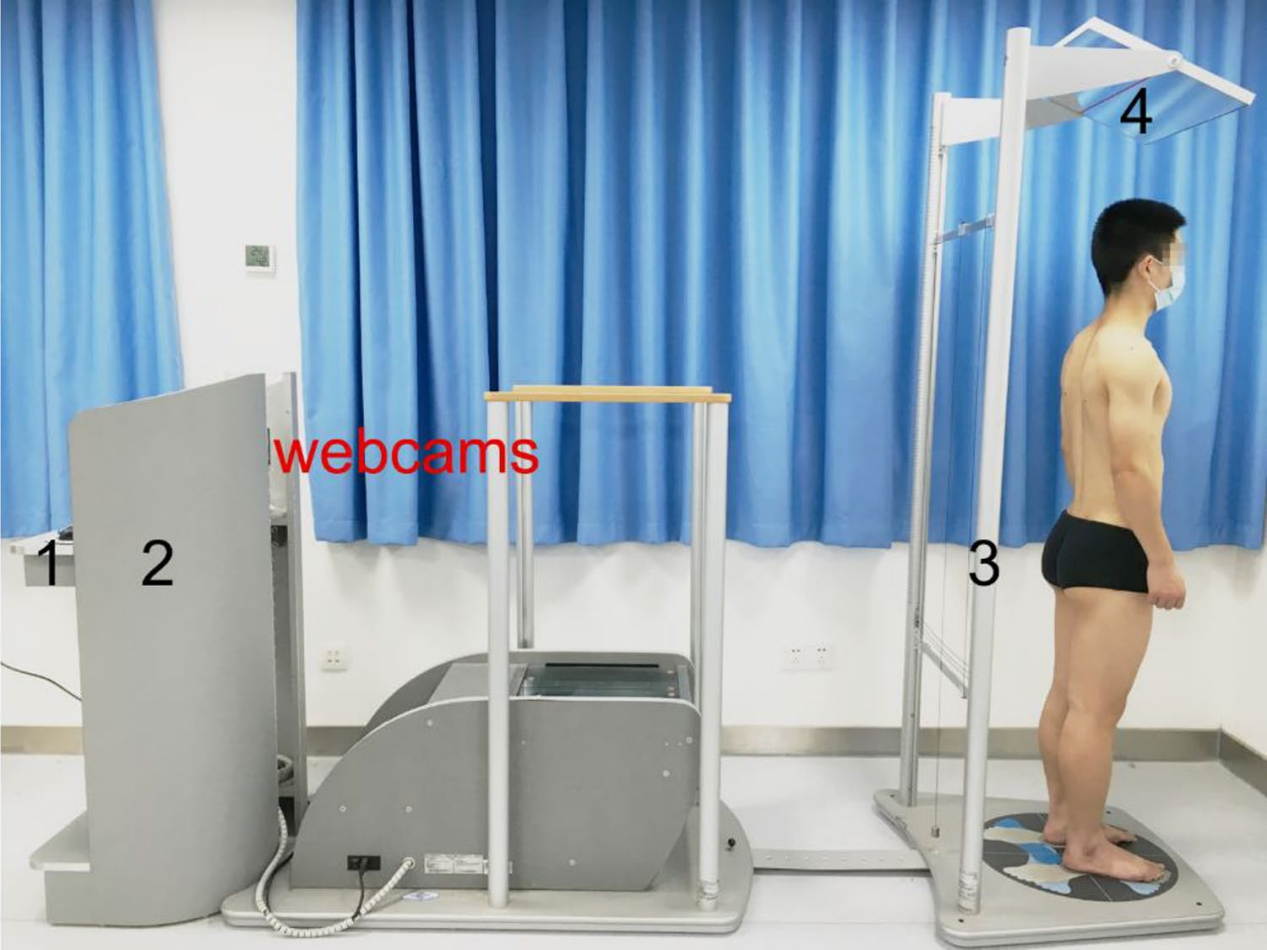
Set-up of recording digital images of participants’ whole body via the 2D photogrammetry method using the GPS 400 posture evaluation system.

The standing posteroanterior full-spine radiographs were taken by a General Radiographic System (uDR780ipro, Shanghai United Imaging Healthcare Co., Ltd., Shanghai, China). The GPS 400 posture analysis system (Chinesport, Italy) that based on the photographic technique was used for posture assessment. The system comprised of the following hardware units: (1) a desktop computer with the modular GPS 5.0 software, (2) a stabilometric platform with webcams (Carl Zeiss Tessar HD 1080P), (3) a posture analysis device with vertical/horizontal strings for postural reference (LUX POSTURAL ANALYZER), and (4) a mirror at the top/superior side of patient (Fig. 1). The height of the webcams was 108 cm, and the distance between the webcams and the posture analysis device was 240 cm. This system can take and record the digital image illustrating the posterior view of the participant’s whole body.

### Leg length discrepancy assessment

The leg length discrepancies were assessed during the participants’ first visits. The leg length discrepancy was defined as the difference between the bilateral leg lengths (i.e., the length from the greater trochanter to lateral ankle). It was manually obtained using the measurement tape when the participants keep relaxed and lying on the examination bad.

### Angle of trunk rotation assessment (ATR)

The angle of trunk rotation (ATR) was measured with a scoliometer for the quantitative assessment of the angle of thoracic rotation, angle of lumbar rotation, and angle of thoracolumbar rotation during the Adam’s test^24^. The Adam’s test was performed with the participant’s feet placed together, knees straight, while bending at the hips to nearly 90° with the arms freely hanging forward and palms together^25^.

### Radiological characteristics

**Figure 2:**
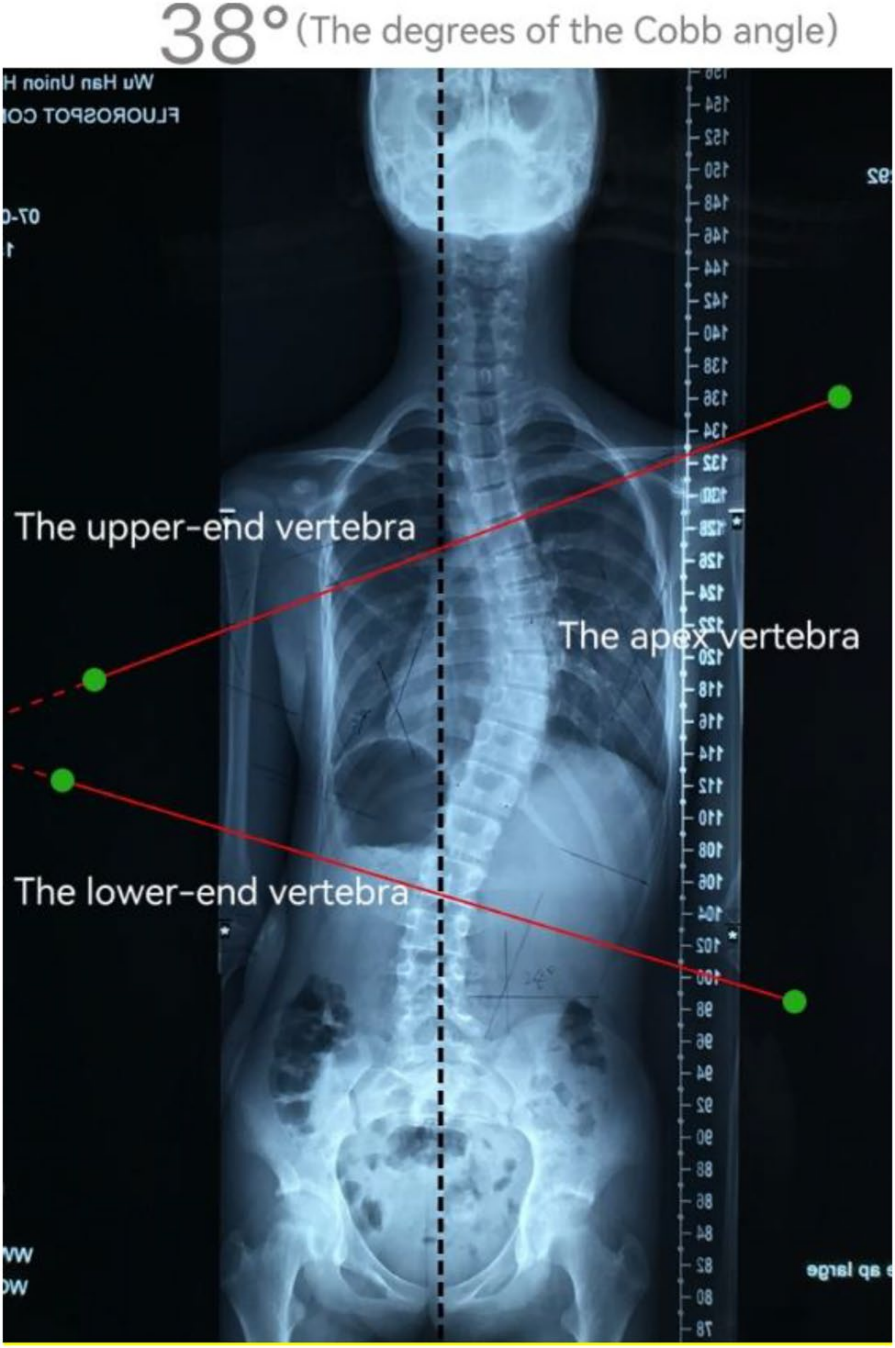
The Cobb angle measured by the iPhone software/application (APP) “Scoliosis Tools” (in this standing posteroanterior full-spine radiograph: the upper-end vertebra is T5, the apex vertebra is T8, the lower-end vertebra is L2, and the degrees of the Cobb angle is 38°).

On each AIS patient’s standing posteroanterior full-spine radiograph, an experienced orthotist conducted the measurement using the iPhone software/application (APP) “Scoliosis Tools”, and the Cobb angle and scoliotic curvature location were recorded (Fig. 2). The radiological characteristics were measured as followed:

1. Cobb angle of the scoliotic curvature (assessed by measuring the angle of the spine curvature between the most tilted upper and lower end vertebrae of a scoliotic curve) (Fig. 2).
2. Scoliotic curvature location (the location of the apex vertebrae) (Fig. 2).

For the radiographic characteristics, the curve types of the participants were divided into single curve (or “C-shape”) and double curves (or “S-shape”)^1^. The location of the curves was classified using the nomenclature proposed by the Scoliosis Research Society (SRS): a curve with an apex between the 2nd and the 11th thoracic vertebrae (T2–T11) is considered as a thoracic (“T”) curve, a curve with an apex at the 12th or 1st lumbar segment (T12–L1) is considered as a thoracolumbar (“TL”) curve, and a curve with an apex between the 2nd and 4th lumbar vertebrae (L2–L4) is considered as a lumbar (“L”) curve^1^.

### Postural indexes

**Figure 3:**
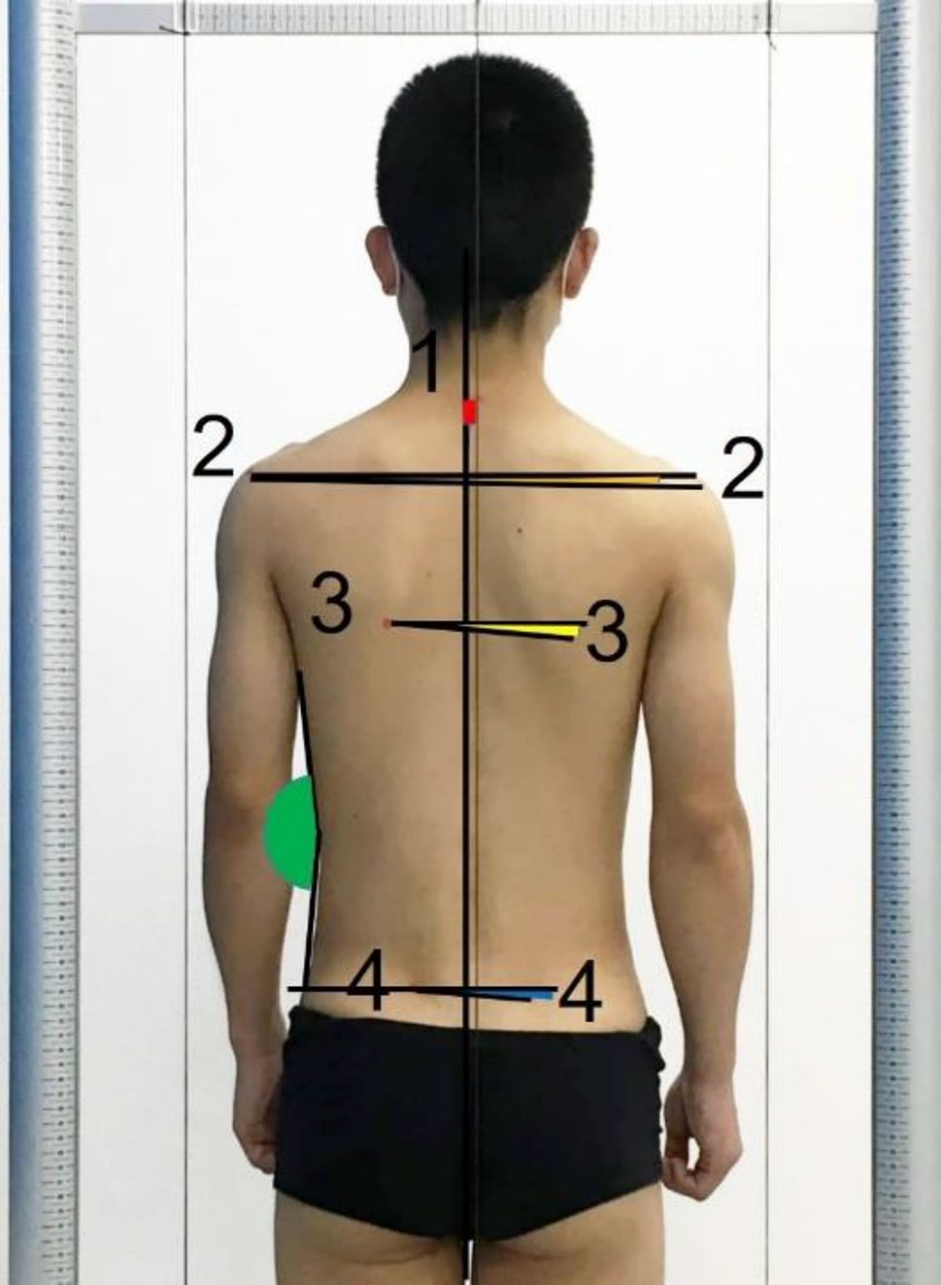
The anatomical mark points (showed by numbers) and the postural indexes (showed by different colored areas).

An experienced physical therapist carried out the assessment of postural indexes for each participant, using the GPS 400 posture analysis system, with the same procedure (Supplementary Appendix 1). Table 1 and Fig. 3 showed the 7 anatomical mark points and the methods for angle and distance calculations of the 5 postural indexes.

**Table 1:** The assessment of postural indexes.

View	Postural landmarks	Postural indexes	Anatomic landmarks
Posterior	(1) C7	1) C7 deviation	(1) Distance between C7 and virtual plumb line across the gluteal cleft (red area)^26^
	(2) Bilateral posterior angle of acromial process	(2) Shoulder alignment	(2) Angle between the line connecting the bilateral posterior angle of acromion processes and the horizontal reference line (orange area)^14^
	(3) Bilateral inferior angle of scapula	(3) Scapula alignment	(3) Angle between the lines connecting the bilateral inferior angle of scapula and the horizontal line (yellow area)^14^
		(4) Waist angle discrepancy*	(4) Difference between the right and left sides’ angles between the line from the axilla to the deepest waist crease and the line from the deepest waist crease to the intersection of the virtual horizontal line of the PSIS and the waist (green area)^13^
	(4) Bilateral PSIS	(5) PSIS alignment	(5) Angle between the line connecting both PSIS and the horizontal line (blue area)^14^

*C7* the 7th cervical vertebra; *PSIS* posterior superior iliac spines.

*Waist discrepancy was the absolute value of the difference between the left and right waist angles.

### Data and statistical analysis

The IBM SPSS (version 23) was used for statistical analysis. The Quantile–Quantile plot method was used to test the normality of each of the calculated variables. Descriptive analyses were conducted to examine the demographic and radiological characteristics of the participants in the study. Intraclass correlation (ICC) values of the inter-rater reliability of the five quantitative postural indexes were calculated using a two-way mixed effects model for single measurement and absolute agreement [i.e., ICC (3,1) model]. The minimal detectable change (MDC) values were calculated using the following equations^27^: SEM = SD × $\sqrt{1-ICC}$, MDC = SEM × 1.96 × $\sqrt{2}$ (SEM: standard error of measurement; SD: standard deviation; ICC: intra-class correlation; MDC: minimal detectable change). The values of the five postural indexes for the following analyses were selected by coin method by the two raters. One-way ANOVA with post hoc Tukey HSD (satisfy the homogeneity of variances) or Games–Howell (do not satisfy the homogeneity of variances) analysis was used to analysis the posterior postural characteristics/indexes measured by 2D photogrammetry of participants in scoliosis screening by comparing the differences between the AIS participants with the Cobb angle between 10° and 45° (defined as “scoliosis group”)^1^ and those with the angle of trunk rotation (ATR) appearing in the Adam’s test measured by the Scoliometer been less than 4° (defined as “non-scoliosis group”). Pearson coefficients of correlation were analyzed to identify any relationships between the thoracic Cobb angle, lumbar Cobb angle of the double-curve/S-shape group; the thoracic Cobb angle, thoracolumbar Cobb angle, lumbar Cobb angle of the single-curve/C-shape group; and each of the five postural indexes, separately. The level of significance was set as 0.05.

### Ethics approval

This study was approved by the Medical Ethics Committee of Tongji Medical College, Huazhong University of Science and Technology (protocol code: [2020] (S173); date of approval: 8th Jul 2020).

## Results

### Demographic and radiological characteristics of the participants

In the progress of retrospectively collecting the participants from the outpatient clinic (specializing in the treatment of scoliosis) from July 2016 to June 2023, a total of 297 subjects with the assessment results of the 2D photogrammetry of the posterior views of the whole body in standing position were initially collected. Among these subjects, 187 cases were excluded according to the inclusion criteria, including 74 cases under 10 years old or over 16 years old; 36 cases with leg length discrepancies; and 77 cases without the assessments results of the standing posteroanterior full-spine radiographs. Finally, a total of 110 participants without leg length discrepancies were involved and analyzed for this study. As shown in Table 2, among the 110 participants, there were 32 cases in single-curve group (13 ± 1 years, 156 ± 8 cm, 47 ± 12 kg, 19 ± 3 kg/m^2^, Cobb angle: 27 ± 10 degrees), 31 cases in double-curve group (13 ± 1 years, 156 ± 8 cm, 47 ± 9 kg, 19 ± 3 kg/m^2^, Thoracic Cobb angle: 31 ± 7 degrees, Lumbar Cobb angle: 29 ± 7 degrees) and 47 cases in the non-scoliosis group (12 ± 1 years, 156 ± 8 cm, 46 ± 9 kg, 19 ± 3 kg/m^2^). Except for significant differences in age among the three groups of participants (p < 0.01), there were no significant differences in height, weight, and BMI values among the three groups.

**Table 2:** Demographic and radiological characteristics of the participants in the study.

Variables	AIS patients’ group		Non-scoliosis group (mean ± SD)	*p* value
	Single-curve group (mean ± SD)	Double-curve group (mean ± SD)		
Age (years)	13 ± 1	13 ± 1	12 ± 1	< 0.01**
Sex (male/female)	11/21	8/23	24/23	N/A
Height (cm)	156 ± 8	156 ± 8	156 ± 8	0.97
Weight (kg)	47 ± 12	47 ± 9	46 ± 9	0.87
BMI (kg/m^2^)	19 ± 3	19 ± 3	19 ± 3	0.94
Cobb angle (°)	27 ± 10 (range 12°–45°)	T, 31 ± 7 (range 14°–45°); L, 29 ± 7 (range 16°–45°)	N/A	N/A
Curvature location	12 T, 7TL, 13L	All TR-LL	N/A	N/A

*SD* standard deviation, *BMI* body mass index, *T* thoracic curves, *TL* thoracolumbar curves, *L* lumbar curves, *TR-LL* right thoracic curves accompanied with left lumbar curves, *N/A* not applicable.

*Statistical difference (p < 0.05).

**Statistical difference (p < 0.01).

### Reliability of the 2D photogrammetry measurement

As illustrated in Table 3, the inter-rater reliability of all postural indexes measured by rater A and rater B ranged from 0.80 to 0.93 (95% confidence interval: 0.72–0.95) in this study. The minimal detectable change (MDC) values in the inter-rater measurement were 0.72 cm, 1.54°, 2.77°, and 5.44° for the C7 deviation, shoulder alignment, scapula alignment, and waist angle discrepancy, respectively.

**Table 3:** Reliability and the MDC values of all postural indexes measured by rater A and rater B.

Postural indexes	Inter-rater reliability	
	ICC (95%CI)	MDC
C7 deviation	0.89 (0.85–0.92)	0.72 cm
shoulder alignment	0.85 (0.79–0.89)	1.54°
scapula alignment	0.87 (0.82–0.91)	2.77°
waist angle discrepancy	0.93 (0.89–0.95)	5.44°
PSIS alignment	0.80 (0.72–0.85)	2.51°

*ICC* intraclass correlation coefficient, *CI* confidence interval, *MDC* minimal detectable change.

### Clinical value of the measured postural indexes in scoliosis screening (i.e., scoliosis group vs. non-scoliosis group)

As shown in Table 4, the AIS participants were divided into three groups: (1) single-curve/C-shape group with Cobb angle been greater than 10° (defined as “single-curve/C-shape scoliosis group/participants”, n = 32), (2) double-curve/S-shape group with Cobb angle been greater than 10° (defined as “double-curve/S-shape scoliosis group/participants”, n = 31), and (3) non-scoliosis group with the angle of trunk rotation (ATR) appearing in the Adam’s test measured by the Scoliometer been smaller than 4° (defined as “non-scoliosis group/participants”, n = 47). The C7 deviation (F [2,107 = 3.5, *p* = 0.03), shoulder alignment (Welch F [2,50.4 = 12.0, *p* < 0.001), scapula alignment (Welch F [2,45.9] = 16.1, *p* < 0.001), waist angle discrepancy (Welch F [2,41.6] = 24.8, *p* < 0.001), and PSIS alignment (Welch F [2,52.5] = 8.7, *p* < 0.001) were significantly different among the three groups.

**Table 4:** Comparison of the posture indexes between scoliosis group and non-scoliosis group.

Postural indexes	Differences between non-scoliosis group and scoliosis group							
	F/Welch F^a^	*p* value	Tukey HSD analysis/Game–Howell analysis					
			C group(n = 32)/N group (n = 47) Mean difference (95% CI)	*p*	S group (n = 31)/N group (n = 47) Mean difference (95% CI)	*p*	S group (n = 31)/C group (n = 32) Mean difference (95% CI)	*p*
C7 deviation	**F (2,107) = 3.5**	0.03*	**0.4 (0.04 to 0.8)**	0.02*	0.1(− 0.3 to 0.5)	0.8	− 0.3 (− 0.8 to 0.1)	0.1
shoulder alignment	**Welch F (2,50.4) = 12.0**	0.001**	0.9 (− 0.01 to 1.8)	0.06	**1.1 (0.5 to 1.7)**	0.001**	0.2 (− 0.7 to 1.2)	0.8
scapula alignment	**Welch F (2,45.9) = 16.1**	0.001**	**3.0 (1.5 to 4.5)**	0.001**	**2.0 (0.5 to 3.4)**	0.04*	− 1.0 (− 2.9 to 0.9)	0.4
waist angle discrepancy	**Welch F (2,41.6) = 24.8**	0.001**	**8.8 (4.6 to 13.0)**	0.001**	**7.0 (3.5 to 10.5)**	0.001**	− 1.8 (− 7.1 to 3.4)	0.6
PSIS alignment	**Welch F (2,52.5) = 8.7**	0.001**	**1.5 (0.3 to 2.8)**	0.01*	**1.1 (0.3 to 2.0)**	**0.05**	− 0.4 (− 1.8 to 1.0)	0.7

*CI* confidence interval, *C group* the single-curve/C-shape group, *S group* the double-curve/S-shape group, *N group* non-scoliosis group.

*Statistical difference (*p* < 0.05).

**Statistical difference (*p* < 0.01).

^a^Results from a 1-way ANOVA analysis.

Significant values are in bold.

For AIS participants with single-curve/C-shape curve, the scoliosis participants have significantly 0.4 cm larger C7 deviation (95% CI 0.04–0.8, *p* = 0.02), 3.0° larger scapula alignment (95% CI 1.5–4.5, *p* < 0.001), 8.8° larger waist angle discrepancy (95% CI 4.8–13.0, *p* < 0.001), and 1.5° larger PSIS alignment (95% CI 0.3–2.8, *p* = 0.01) than the non-scoliosis participants.

For AIS participants with double-curve/S-shape curve, the scoliosis patients have significantly 1.1° larger shoulder alignment (95% CI 0.5–1.7, *p* < 0.001), 2.0° larger scapula alignment (95% CI 0.5–3.4, *p* = 0.04), 7.0° larger waist angle discrepancy (95% CI 3.5–10.5, *p* < 0.001), and 1.1° larger PSIS alignment (95% CI 0.3–2.0, *p* = 0.05) than the non-scoliosis participants.

Based on the comparison of the inter-rater minimal detectable change (MDC) values (Table 3), it was found that the differences of the scapula alignment (3.0°) and the waist angle discrepancy (8.8°) between the single-curve/C-shape scoliosis group and the non-scoliosis group exceeded the inter-rater minimal detectable change (MDC) values (2.77° for the scapula alignment and 5.44° for the waist angle discrepancy), but the estimates were too imprecise to exclude the possibility that the effect is trivial for the scapula alignment (95% CI 1.5–4.5) and for the waist angle discrepancy (95% CI 4.8–13.0). Additionally, the difference of the waist angle discrepancy (7°) between the double-curve/S-shape curve group and the non-scoliosis group exceeded the inter-rater minimal detectable change (MDC) values (5.44° for the waist angle discrepancy), but the estimate was also too imprecise to exclude the possibility that the effect is trivial for the waist angle discrepancy (95% CI 3.5–10.5). Meanwhile, the remaining differences of the C7 deviation (0.4 cm), the shoulder alignment (1.1°), scapula alignment (2°) and the PSIS alignment (1.5° and 1.1°) between the non-scoliosis group and the single-curve/C-shape or double-curve/S-shape curve group did not exceed the inter-rater minimal detectable change (MDC) values (0.72 cm for the C7 deviation, 1.54° for the shoulder alignment, 2.77° for the scapula alignment, 5.44° for the waist angle discrepancy, and 2.51° for the PSIS alignment).

No statistic difference existed between double-curve/S-shape curve scoliosis group/participants and single-curve/C-shape scoliosis group/participants in any of the five postural indexes in this study.

### Clinical value of the measured postural indexes in scoliosis monitoring (i.e., correlation between Cobb angles and postural indexes)

As shown in Table 5, among all the three types of Cobb angles and in both scoliosis groups, the waist angle discrepancy had moderate to strong positive correlation with the lumbar or thoracolumbar Cobb angle for both the single-curve and the double-curve group (r = 0.4, *p* = 0.01; r = 0.8, *p* = 0.03; r = 0.7, *p* = 0.01). The shoulder alignment had moderate positive correlation with the thoracic Cobb angle of the single-curve group (r = 0.6, *p* = 0.03). No statistical difference existed in the remaining correlations between the postural indexes and the Cobb angles of the participated scoliosis patients.

**Table 5:** Correlation between the posture indexes and Cobb angles.

Postural indexes	Location/type of Cobb angles									
	T^s^ (n = 31)		L^s^ (n = 31)		T^c^ (n = 12)		TL^c^ (n = 7)		L^c^ (n = 13)	
	r^a^	*p*	r	*p*	r	*p*	r	*p*	r	*p*
C7 deviation	− 0.01	0.9	0.2	0.2	0.4	0.2	0.4	0.4	0.5	0.1
Shoulder alignment	0.1	0.5	0.1	0.6	**0.6**	0.03*	− 0.4	0.4	0.3	0.3
Scapula alignment	− 0.03	0.8	0.1	0.6	0.4	0.2	− 0.2	0.7	0.3	0.3
Waist angle discrepancy	0.06	0.8	**0.4**	0.01*	0.4	0.2	**0.8**	0.03*	**0.7**	0.01*
PSIS alignment	0.1	0.8	0.3	0.1	0.4	0.2	0.4	0.4	0.2	0.5

*Statistical difference (*p* < 0.05).

**Statistical difference (*p* < 0.01).

^a^Results from Pearson coefficients of correlation analysis.

T^s^: The thoracic Cobb angle of the double-curve/S-shape group.

L^s^: The lumbar Cobb angle of the double-curve/S-shape group.

T^c^: The thoracic Cobb angle of the single-curve/C-shape group.

TL^c^: The thoracolumbar Cobb angle of the single-curve/C-shape group.

L^c^: The lumbar Cobb angle of the single-curve/C-shape group.

Significant values are in bold.

## Discussion

This study has developed, applied, and evaluated a novel protocol of measuring five postural indexes by 2D photogrammetry method in AIS patients with mild to moderate curvatures and receiving conservative treatments; and identified that the C7 deviation, shoulder alignment, scapula alignment, waist angle discrepancy, and PSIS alignment could screen and monitor the scoliotic curvatures of AIS patients.

### Reliability of the 2D photogrammetry measurement

The inter-rater reliability of the five postural indexes measured by rater A and rater B was moderate to high (0.80 to 0.93 [95% CI 0.72–0.95]) in this study. This finding has been in line with the previous study which investigated the overall test–retest and inter-rater reliability of posture indexes among patients with idiopathic scoliosis, and reported a good level of reliability (ICC: 0.67–0.99) for 26 posture indexes out of 32 with 49 anatomical mark points^15^. In this study, the further reduced number of 7 anatomical mark points for 5 posture indexes could decrease the assessment time and the bias derived from palpation, which has been important to improve the reliability of the assessments based on photograph analysis^28^. The moderate to high inter-rater reliability of the novel postural-index measurement in this study also supported the feasibility and reliability of employing this protocol in future research and clinical practice, with further optimizations.

### Clinical value of the measured postural indexes in scoliosis screening (i.e., scoliosis group vs. non-scoliosis group)

While the previous AIS assessments have the concerns of radiological hazards^3^, this study has identified the possibility of several postural indexes that could be used to screen and identify the AIS patients with Cobb angles larger than 10°. More specifically, this study has observed that the postural indexes of the C7 deviation, shoulder alignment, scapula alignment, waist angle discrepancy and PSIS alignment could identify the AIS patients with Cobb angles been larger than 10°, from the non-scoliosis participants. This finding could build on the previous knowledge on scoliosis screening, by identifying and validating more postural indexes measured by the 2D photogrammetry method. The previous study has mixed the AIS patients with single- and double-curve types for data analysis, which may partially explain why the postural index (i.e., the shoulder obliquity) identified for routine scoliosis assessment^14^ was not consistent with the postural indexes in this study (i.e., the C7 deviation, shoulder alignment, scapula alignment, waist angle discrepancy and PSIS alignment). Upon improving the participant homogeneity and control of the confounding factors by analyzing the postural indexes based on the specific curvature type of AIS patients, this study has identified that the C7 deviation, shoulder alignment, scapula alignment, waist angle discrepancy and PSIS alignment can be considered for scoliosis screening, and has potentially improved the reliability and accuracy of the scoliosis screening. Meanwhile, considering the comparison results of the inter-rater minimal detectable change (MDC) values and the differences between the non-scoliosis group and the single-curve/C-shape or double-curve/S-shape curve group of this study, further multi-center clinical trials with large sample sizes can be conducted to further verify such findings before clinical implementation.

In screening programs for children and adolescents, the Adam’s forward bending tests combining with/without the angle of trunk inclination (ATI, or ATR/Angle of Trunk Rotation), as measured by the Scoliometer, are the main tests in the clinical examination^1^. However, the forward bending test alone has been considered as insufficient^21^. Yet, the forward bending test with the Scoliometer measurement has been found with lower inter-rater reliabilities than the intra-rater ones for the same spinal segments of upper and low thorax and lumbar spine, even when the errors from palpation and positioning of the instrument were eliminated^29^. Considering that the 2D photogrammetry method can reduce the difficulty of subjects’ cooperation and minimize the errors caused by poor cooperation, the identified five postural indexes of this study can be considered in future clinical practice to supplement the existing screening methods. No statistical difference was found between double-curve/S-shape scoliosis participants and the single-curve/C-shape scoliosis participants for each of the five postural indexes in this study. This finding is different from previous research findings that the trunk posture indexes varied in scoliosis patients with different scoliotic curvature types^13, 18^. The possible reason for such different finding could be due to the different classification methods, i.e., the Rigo classification^18^ and the Lenke classification^13^ used in the previous studies were more detailed than the classification method of double-curve/S-shape or single-curve/C-shape used in the current study. Future clinical practice shall pay attention to this, and use the classification method of double-curve/S-shape or single-curve/C-shape when assessing AIS patients using the introduced postural indexes obtained through 2D photogrammetry method in this paper.

### Clinical value of the measured postural indexes in scoliosis monitoring (i.e., correlation between Cobb angles and postural indexes)

This study identified that the waist angle discrepancy and shoulder alignment have moderate to strong positive correlations with the Cobb angle, supporting that these postural indexes could be used to monitor the scoliotic curvature changes in AIS patients. Considering that the risk of curvature progression is high until reaching skeletal maturity^30^, the routine and regular monitoring of the scoliotic curvature changes is essential for improving the treatment efficiency in AIS patients. Recent studies have also shown that the scoliosis-specific exercises focusing on postural rehabilitation can improve the spinal curvatures in growing AIS adolescents with positive outcomes^31^. By initially identifying several 2D-photogrammetry measured postural indexes which have moderate to good correlation with Cobb angles, this pilot study has preliminarily provided a new, radiation-free, quick, easy, inexpensive, and accessible approach of monitoring the changes of scoliosis curve during the treatment and follow-ups of AIS patients. Further studies could also consider combining the postural indexes of the waist angle discrepancy and shoulder alignment together, and develop a formula that can be used to monitor the progression of scoliotic curvature in AIS patients, and reduce the high referral rate of receiving radiological assessment in AIS patients^3^. It is expected that the results of such formula shall be in line with the progression of scoliotic curvature in AIS patients.

The aesthetic self-perception and appearance have tended to affect the AIS patient’s quality of life and visual correction of scoliosis-related external trunk deformity, and have been an important issue for AIS patients during conservative treatment^1^. To date, the main outcome measures concerning the aesthetic effects of treatment have been questionnaires^32^. When the study aim is to evaluate the perceived self-image in patients, the drawing-based questionnaires may be the optimal choice^33^. It shall be feasible to apply some of the identified 2D-photogrammetry measured postural indexes to feedback on the aesthetic and appearance changes for the AIS patients in future practice. Further studies and efforts are still needed to determine whether the positive feedback of appearance could help improve the quality of life of AIS patients or not.

### Future research outlook

With the advancement in imaging processing and recognition technologies, future efforts can also be put on integrating the automatic recognition of surface anatomical landmarks of AIS patients^34^, which could improve the assessment efficiency and facilitate the screening and monitoring of scoliosis progression in community-/home-based settings in the future. More efforts and studies are still needed to address such needs in the future.

### Limitation

The sample size of this study was not large. More studies with larger sample sizes and multiple centers are needed to solidify the findings reported in this study in the future. Due to the limited sample size, the comparisons of more detailed curve types with different curve locations, curve directions, and curve numbers (e.g., the single left thoracic curves varus single left thoracolumbar curves etc.) were lacked in this study.

## Conclusion

This study has developed a novel method and identified good inter-rater reliability of five postural index (C7 deviation, shoulder alignment, scapula alignment, waist angle discrepancy and PSIS alignment) in screening and two postural indexes (waist angle discrepancy and shoulder alignment) in monitoring the scoliotic curvature changes in AIS patients receiving conservative treatments. This could support the clinical value of this new assessment method, and inspire the future research and clinical practice.

### Supplementary Information

Supplementary Information.

## Data Availability

The data presented in this study are available on request from the corresponding author.

## References

[1] Negrini S, Donzelli S, Aulisa AG, Czaprowski D, Schreiber S, de Mauroy JC (2018). 2016 SOSORT guidelines: Orthopaedic and rehabilitation treatment of idiopathic scoliosis during growth. Scoliosis Spinal Disord..

[2] Zheng Y, Dang Y, Wu X, Yang Y, Reinhardt JD, He C (2017). Epidemiological study of adolescent idiopathic scoliosis in Eastern China. J. Rehabil. Med..

[3] Altaf F, Drinkwater J, Phan K, Cree AK (2017). Systematic review of school scoliosis screening. Spine Deformity.

[4] Trac S, Zheng R, Hill DL, Lou E (2019). Intra- and interrater reliability of Cobb angle measurements on the plane of maximum curvature using ultrasound imaging method. Spine Deformity.

[5] Roye BD, Simhon ME, Matsumoto H, Bakarania P, Berdishevsky H, Dolan LA (2020). Establishing consensus on the best practice guidelines for the use of bracing in adolescent idiopathic scoliosis. Spine Deformity.

[6] Yan B, Lu X, Qiu Q, Nie G, Huang Y (2020). Association between incorrect posture and adolescent idiopathic scoliosis among Chinese adolescents: Findings from a large-scale population-based study. Front. Pediatr..

[7] Negrini S, Donzelli S, Di Felice F, Zaina F, Caronni A (2020). Construct validity of the Trunk Aesthetic Clinical Evaluation (TRACE) in young people with idiopathic scoliosis. Ann. Phys. Rehabil. Med..

[8] Wang L, Xia N, Wang C, Zheng Q, Ma CZ, Youssef ASA (2022). Optimized scheme for paired transverse corrective forces in S-shaped scoliosis via ultrasound and application in Cheneau brace: A pilot study. Prosthet. Orthot. Int..

[9] Lou E, Nguyen D, Hill D, Raso J (2021). Validation of a novel handheld 3D ultrasound system for imaging scoliosis—Phantom study. Stud. Health Technol. Inform..

[10] Applebaum A, Nessim A, Cho W (2020). Understanding breast asymmetry and its relation to AIS. Spine Deformity.

[11] Fortin C, Feldman DE, Cheriet F, Labelle H (2010). Validity of a quantitative clinical measurement tool of trunk posture in idiopathic scoliosis. Spine.

[12] Levi D, Springer S, Parmet Y, Ovadia D, Ben-Sira D (2019). Acute muscle stretching and the ability to maintain posture in females with adolescent idiopathic scoliosis. J. Back Musculoskelet. Rehabil..

[13] Bago J, Pizones J, Matamalas A, D’Agata E (2019). Clinical photography in severe idiopathic scoliosis candidate for surgery: Is it a useful tool to differentiate among Lenke patterns?. Eur. Spine J..

[14] Penha PJ, Penha NLJ, De Carvalho BKG, Andrade RM, Schmitt ACB, Joao SMA (2017). Posture alignment of adolescent idiopathic scoliosis: Photogrammetry in scoliosis school screening. J. Manipulat. Physiol. Ther..

[15] Fortin C, Feldman DE, Cheriet F, Gravel D, Gauthier F, Labelle H (2012). Reliability of a quantitative clinical posture assessment tool among persons with idiopathic scoliosis. Physiotherapy.

[16] Prowse A, Pope R, Gerdhem P, Abbott A (2016). Reliability and validity of inexpensive and easily administered anthropometric clinical evaluation methods of postural asymmetry measurement in adolescent idiopathic scoliosis: A systematic review. Eur. Spine J..

[17] Aroeira RM, de Las Casas EB, Pertence AE, Greco M, Tavares JM (2016). Non-invasive methods of computer vision in the posture evaluation of adolescent idiopathic scoliosis. J. Bodyw. Mov. Ther..

[18] Rigo MD, Villagrasa M, Gallo D (2010). A specific scoliosis classification correlating with brace treatment: Description and reliability. Scoliosis.

[19] Hamada T, Matsubara H, Kato S, Hikichi T, Shimokawa K, Demura S, Tsuchiya H (2022). Correlation analysis between leg-length discrepancy and lumbar scoliosis using full-length standing radiographs. Strateg. Trauma Limb Reconstr..

[20] Sheha ED, Steinhaus ME, Kim HJ, Cunningham ME, Fragomen AT, Rozbruch SR (2018). Leg-length discrepancy, functional scoliosis, and low back pain. JBJS Rev..

[21] Fong DY, Lee CF, Cheung KM, Cheng JC, Ng BK, Lam TP, Mak KH, Yip PS, Luk KD (2010). A meta-analysis of the clinical effectiveness of school scoliosis screening. Spine.

[22] Papaioannou T, Stokes I, Kenwright J (1982). Scoliosis associated with limb-length inequality. J. Bone Jt. Surg..

[23] Negrini S, Hresko TM, O’Brien JP, Price N (2015). Recommendations for research studies on treatment of idiopathic scoliosis: Consensus 2014 between SOSORT and SRS non-operative management committee. Scoliosis.

[24] Côté P, Kreitz BG, Cassidy JD, Dzus AK, Martel J (1998). A study of the diagnostic accuracy and reliability of the scoliometer and Adam’s forward bend test. Spine.

[25] Bunnell WP (1984). An objective criterion for scoliosis screening. J. Bone Jt. Surg. Am..

[26] Fortin C, Grunstein E, Labelle H, Parent S, Ehrmann Feldman D (2016). Trunk imbalance in adolescent idiopathic scoliosis. Spine J..

[27] Saner RJ, Washabaugh EP, Krishnan C (2017). Reliable sagittal plane kinematic gait assessments are feasible using low-cost webcam technology. Gait Posture.

[28] Ferreira EA, Duarte M, Maldonado EP, Bersanetti AA, Marques AP (2011). Quantitative assessment of postural alignment in young adults based on photographs of anterior, posterior, and lateral views. J. Manipulat. Physiol. Ther..

[29] Bonagamba GH, Coelho DM, Oliveira AS (2010). Inter and intra-rater reliability of the scoliometer. Rev. Bras. Fisioter.

[30] Johnson MA, Flynn JM, Anari JB, Gohel S, Cahill PJ, Winell JJ (2021). Risk of scoliosis progression in nonoperatively treated adolescent idiopathic scoliosis based on skeletal maturity. J. Pediatr. Orthop..

[31] Weiss HR, Moramarco MM, Borysov M, Ng SY, Lee SG, Nan X (2016). Postural rehabilitation for adolescent idiopathic scoliosis during growth. Asian Spine J..

[32] Auerbach JD, Lonner BS, Crerand CE, Shah SA, Flynn JM, Bastrom T, Penn P, Ahn J, Toombs C, Bharucha N, Bowe WP (2014). Body image in patients with adolescent idiopathic scoliosis. J. Bone Jt. Surg. Am..

[33] Babaee T, Moradi V, Shariat A, Anastasio AT, Khani A, Bagheri M (2022). Disease-specific outcome measures evaluating the health-related quality of life of children and adolescents with idiopathic scoliosis and Scheuermann’s kyphosis: A literature review. Spine Surg. Relat. Res..

[34] Michoński J, Glinkowski W, Witkowski M, Sitnik R (2012). Automatic recognition of surface landmarks of anatomical structures of back and posture. J. Biomed. Opt..

