# Supplementary material for: A feasibility study of applying two-dimensional photogrammetry for screening and monitoring of patients with adolescent idiopathic scoliosis in clinical practice
Source: Sci Rep. 2023 Aug 31;13:14273. doi: 10.1038/s41598-023-41267-2 (PMC10471746; doi:10.1038/s41598-023-41267-2)
Supplement: Supplementary file 1 — Supplementary Information. [file 41598_2023_41267_MOESM1_ESM.docx]

Appendix 1

The posture analysis procedure:

1) Before each assessment, the physical therapist calibrated the webcam, the GPS 5.0 software, and the vertical/horizontal strings of the postural analysis device; and informed the patient about the evaluation process;

2) The AIS patient then entered the evaluation room (with appropriate room temperature, i.e., 28℃), while male patients wore underwear and female patients wore underwear and shorts (so the anatomical mark points can be recorded);

3) The patient stood on the mat in a natural and comfortable position, and the assessor posted the dot mark (with a diameter of about 8 mm) on the 7 anatomical mark points [1] (Table 1);

4) After marking down the anatomical landmarks, each patient was instructed to place his/her feet on the posture analysis device;

5) Each AIS patient was then asked to take 5 deep and long breaths in and out, followed by a relaxed standing position. Approximately after 40–60 s, the digital image/photo of the patient was taken in the posterior (by referring to the fixed footprints on the Lux Postural Analyzer), using the webcams of the GPS 400 posture analysis system;

6) Each assessment of one AIS patient was completed within 10 minutes;

7) From the captured digital image, the 5 quantitative postural indexes (Table 1) were calculated by rater A and rater B without interference from each other using the proprietary algorithms of the chosen analysis parameters in the GPS 5.0 software system with the auxiliary plumb lines been drawn on the image during the postural analysis [1, 2].

**Table 1**

The assessment of postural indexes.

| **View** | **Postural landmarks** | **Postural indexes** | **Anatomic landmarks** |
| --- | --- | --- | --- |
| posterior | 1) C7 | 1) C7 deviation | 1) distance between C7 and virtual plumb line across the gluteal cleft [3] |
|  | 2) bilateral posterior angle of acromial process | 2) shoulder alignment | 2) angle between the line connecting the bilateral posterior angle of acromion processes and the horizontal reference line [1] |
|  | 3) bilateral inferior angle of the scapula | 3) scapula alignment | 3) angle between the lines connecting the bilateral inferior angle of the scapula and the horizontal line [1] |
|  |  | 4) waist angle discrepancy * | 4) difference between the right and left sides' angles between the line from the axilla to the deepest waist crease and the line from the deepest waist crease to the intersection of the virtual horizontal line of the PSIS and the waist [2] |
|  | 4) bilateral PSIS | 5) PSIS alignment | 5) angle between the line connecting both PSIS and the horizontal line [1] |

Note: C7: the 7th cervical vertebra; PSIS: posterior superior iliac spines; * Waist discrepancy was the absolute value of the difference between the left and right waist angles.

# References

[1] P.J. Penha, N.L.J. Penha, B.K.G. De Carvalho, R.M. Andrade, A.C.B. Schmitt, S.M.A. Joao, Posture Alignment of Adolescent Idiopathic Scoliosis: Photogrammetry in Scoliosis School Screening, Journal of manipulative and physiological therapeutics 40(6) (2017) 441-451. <https://www.ncbi.nlm.nih.gov/pubmed/28822476>.

[2] J. Bago, J. Pizones, A. Matamalas, E. D’Agata, Clinical photography in severe idiopathic scoliosis candidate for surgery: is it a useful tool to differentiate among Lenke patterns?, European Spine Journal 28(12) (2019) 3018-3025.

[3] C. Fortin, E. Grunstein, H. Labelle, S. Parent, D. Ehrmann Feldman, Trunk imbalance in adolescent idiopathic scoliosis, The spine journal : official journal of the North American Spine Society 16(6) (2016) 687-93. <https://www.ncbi.nlm.nih.gov/pubmed/26921627>.
